# “As long as that place stays open, I’ll stay alive”: Accessing injectable opioid agonist treatment during dual public health crises

**DOI:** 10.1186/s12954-023-00779-w

**Published:** 2023-04-14

**Authors:** Kaitlyn Jaffe, Sarin Blawatt, Eisha Lehal, Kurt Lock, Adam Easterbrook, Scott MacDonald, Scott Harrison, Julie Lajeunesse, David Byres, Martin Schechter, Eugenia Oviedo-Joekes

**Affiliations:** 1 Center for Bioethics and Social Sciences in Medicine, University of Michigan, 2800 Plymouth Road, Ann Arbor, MI 48109 USA; 2 Centre for Health Evaluation and Outcome Sciences, Providence Health Care, St. Paul’s Hospital, 575-1081 Burrard St., Vancouver, BC V6Z 1Y6 Canada; 3 BC Centre for Disease Control, Provincial Health Services Authority, 655 West 12th Avenue, Vancouver, BC V5Z 4R4 Canada; 4 Providence Health Care, Providence Crosstown Clinic, 84 West Hastings Street, Vancouver, BC V6B 1G6 Canada; 5 Provincial Health Services Authority, 200-1333 W Broadway, Vancouver, BC V6H 4C1 Canada; 6 School of Population and Public Health, University of British Columbia, 2206 East Mall, Vancouver, BC V6T 1Z3 Canada

**Keywords:** Injectable opioid agonist treatment, COVID-19 pandemic, Substance use, Canada

## Abstract

**Background:**

Since the onset of the COVID-19 pandemic, overdose rates in North America have continued to rise, with more than 100,000 drug poisoning deaths in the past year. Amidst an increasingly toxic drug supply, the pandemic disrupted essential substance use treatment and harm reduction services that reduce overdose risk for people who use drugs. In British Columbia, one such treatment is injectable opioid agonist treatment (iOAT), the supervised dispensation of injectable hydromorphone or diacetylmorphine for people with opioid use disorder. While evidence has shown iOAT to be safe and effective, it is intensive and highly regimented, characterized by daily clinic visits and provider–client interaction—treatment components made difficult by the pandemic.

**Methods:**

Between April 2020 and February 2021, we conducted 51 interviews with 18 iOAT clients and two clinic nurses to understand how the pandemic shaped iOAT access and treatment experiences. To analyze interview data, we employed a multi-step, flexible coding strategy, an iterative and abductive approach to analysis, using NVivo software.

**Results:**

Qualitative analysis revealed the ways in which the pandemic shaped clients’ lives and the provision of iOAT care. First, client narratives illuminated how the pandemic reinforced existing inequities. For example, socioeconomically marginalized clients expressed concerns around their financial stability and economic impacts on their communities. Second, clients with health comorbidities recognized how the pandemic amplified health risks, through potential COVID-19 exposure or by limiting social connection and mental health supports. Third, clients described how the pandemic changed their engagement with the iOAT clinic and medication. For instance, clients noted that physical distancing guidelines and occupancy limits reduced opportunities for social connection with staff and other iOAT clients. However, pandemic policies also created opportunities to adapt treatment in ways that increased patient trust and autonomy, for example through more flexible medication regimens and take-home oral doses.

**Conclusion:**

Participant narratives underscored the unequal distribution of pandemic impacts for people who use drugs but also highlighted opportunities for more flexible, patient-centered treatment approaches. Across treatment settings, pandemic-era changes that increase client autonomy and ensure equitable access to care are to be continued and expanded, beyond the duration of the pandemic.

## Introduction

In North America, over 100,000 people have died from drug poisoning in the past year, due to the increasingly toxic drug supply [1, 2]. Compounding this, the COVID-19 pandemic increased overdose risks for people who use drugs (PWUD), in part through reductions in the availability or access to essential health and treatment services [3–5]. Among those essential services, opioid agonist treatment (OAT), including methadone and buprenorphine, have been demonstrated as safe and effective for the treatment of opioid use disorder [6, 7]. Over the past several decades, advances have been made to expand medication treatment options to include injectable opioid agonist treatment (iOAT), utilized in several European countries and across five Canadian provinces [8–12]. In these settings, when oral OAT is ineffective, clients can access iOAT treatment, which can include diacetylmorphine (i.e., pharmaceutical-grade heroin) or hydromorphone (an acute opioid pain analgesic), although other formulations may become more available (e.g., injectable buprenorphine in depot forms, fentanyl, among others). While shown to be safe and effective [12–15], iOAT is a high intensity treatment, characterized by daily clinic visits, observed self-administration, and significant provider–client interactions—treatment elements that may prove difficult under pandemic conditions of social distancing. In light of these pandemic-era challenges, we conducted 51 qualitative interviews with 18 clients accessing iOAT in Vancouver and two clinic nurses, to understand how the pandemic shaped clients’ perceptions of and access to iOAT in a rapidly evolving treatment context.

Injectable opioid agonist treatment, in the form of diacetylmorphine or hydromorphone, can be prescribed to people who inject opioids, including those who have not benefitted from other opioid agonist treatments. Typically, in iOAT provision, medications are dispensed at specialized clinics and observed by health providers to ensure safe self-administration by clients and to monitor for adverse effects [15, 16]. Clients generally begin attending iOAT clinic three times per day, doses are individualized, and visits can be reduced or injectable medications combined with oral formulations, in consultation with the clinical team. Previous analyses in Europe and Canada have found iOAT to be associated with reduced use of unregulated opioids, reduced engagement in prohibited or illegal activities, and with improvements in stability and overall wellbeing [12–14]. In the current study, clients were receiving treatment at Canada’s first iOAT clinic, located in Vancouver’s Downtown Eastside, a neighborhood characterized by socioeconomic marginalization, drug markets, and heavy police surveillance, as well as strong community bonds and a large network of health and harm reduction services [17]. There was also one client receiving treatment at a clinic located in Surrey, a city just outside of Vancouver. Emerging research on the perspectives of clients in the community clinic has highlighted strengths of the iOAT program, such as holistic care of clients and reliable access to safe medication, as well as areas for improvement, including rigid and inflexible treatment schedules [18]. However, since the onset of the pandemic, little is known around how iOAT clinics have adapted to COVID-19-related challenges or how clients have responded to evolving clinical practices in the provision of substance use treatment.

Globally, the pandemic significantly interrupted the treatment of substance use disorders, and drew attention to pervasive disparities in treatment access and availability [19–22]. For clients receiving iOAT, the consequences of treatment disruption may be considerable, given the frequency of doses, the intensity of services, and the increasingly toxic drug supply in British Columbia that amplifies overdose risk. Emerging research with Canadian harm reduction and treatment providers during the pandemic has illustrated how staff had to ensure safe access to treatment while navigating provincial restrictions, service interruptions, increased staff responsibilities (e.g., cleaning, viral screening and testing), the development of new protocols, and their own risk of exposure to COVID-19 [23]. Recent work has also demonstrated how PWUD avoided harm reduction services during the pandemic due to COVID-19 risks [24], and how potential patients in British Columbia reduced their utilization of general healthcare services and of transit to healthcare systems during the pandemic [25, 26]. Beyond effects of the virus itself, the pandemic also threatened people’s financial, mental, and physical wellbeing—impacts that are magnified for socioeconomically marginalized PWUD, including iOAT clients, who are more likely to experience financial instability, homelessness, and decreased support for social, physical and mental health needs [4, 27, 28]. However, despite myriad challenges in supporting PWUD and ensuring treatment access, the pandemic also presented new opportunities to develop clinic procedures and treatment protocols that address prior critiques and can be tailored to the needs of iOAT clients [18, 23, 29]. To investigate these complex changes and impacts, we draw on qualitative interviews with clients accessing iOAT and explore how the pandemic shaped their perspectives of and experiences with treatment, as well as make recommendations for providers and policymakers in the future provision of iOAT programs and in supporting iOAT clients.

## Methods

### Study overview

This analysis is a qualitative study nested within a larger multi-methods investigation—the Program of Outcomes Research on Treatment with Injectables for Addiction (PORTIA) study—that aims to understand and improve clients’ and providers’ experiences with injectable opioid agonist treatment (iOAT) in Canada. Currently, the iOAT program offers injectable hydromorphone or diacetylmorphine for eligible clients with opioid use disorder at a community clinic in Vancouver, British Columbia, the first clinic in North America to provide these treatment options [14]. While previous work has discussed clinical recommendations and protocols around iOAT [14, 30], the COVID-19 pandemic required the iOAT clinic to make significant changes to the provision of services. For instance, under the guidance of their doctor, some clients were able to reduce their visit frequency and access take-home oral doses of medication or receive home delivery of oral medication if they were at severe risk of complications from COVID-19 [23]. Take-home injectable doses were not permitted at the time.

### Study design and data collection

From April 2020 to February 2021, the PORTIA study team conducted a series of semi-structured interviews with 18 clients who accessed daily iOAT and two nurses from the clinic. IOAT clients were recruited for participation either through prior involvement with other PORTIA study components, through snowball sampling, or through recommendations from research or clinic staff. Interviewers were trained qualitative researchers and unaffiliated with the clinic. Participants were interviewed at three timepoints to facilitate assessing their treatment experiences and perspectives over the course of the pandemic. A cohort of 18 participants were interviewed between April and May of 2020 (Wave A), again between May and June of 2020 (Wave B), and 13 participants were interviewed in February 2021 (Wave C). Eighteen clients completed at least two interviews and 13 clients completed all three interviews. As discussed by previous assessments of the clinic, iOAT clients have an average age of 45 with a mean of 15 lifetime years of injection opioid use. A majority of clients are living with a chronic illness and have experienced housing instability in the past three years, but retention rates with iOAT remain high [14, 18]. The participant demographic details for this study are listed in Table 1, including gender identity and self-reported race/ethnicity. To provide staff perspectives and help triangulate data, two nurses were interviewed in October 2020.

**Table 1:** PORTIA participant demographics

Characteristic	*n* (%) (*N* = 18)
Age^†^	
Under 40	4 (22.2)
40–60	9 (50.0)
60–80	3 (16.7)
Gender	
Men	8 (44.4)
Women	10 (55.6)
Race/ethnicity^†^	
White	11 (61.1)
Indigenous ancestry	5 (27.8)

Two clinic nurses were also interviewed

^**†**^Age and race/ethnicity data missing for two participants; percentages do not sum to 100

Interview questions focused on iOAT clients’ experiences with treatment medication and the clinic throughout the pandemic, as well as individual (e.g., health), social (e.g., community relationships) and structural (e.g., economic) characteristics that may have impacted their treatment. Participants were also asked about their perceptions of the pandemic and COVID-19 risks for themselves and their community, and of COVID-19 mitigation measures and government responses. Interviews with nurses focused on how clinic services have changed over the course of the pandemic and how clients and staff responded. Interviews were conducted via Zoom audio calls or over the phone and lasted approximately 30 min. Participants were given $30 CAD for their time and expertise. All PORTIA participants provided either written consent before the onset of physical distancing requirements, or verbal consent. Each interview was recorded, transcribed, and deidentified.

### Data analysis

Prior to the current analysis, members of the study team completed a primary analysis that used a longitudinal, grounded theory approach, focused on processes of connecting and engaging with the iOAT clinic during the ongoing COVID-19 pandemic, which had been presented in conferences during the pandemic [31–33]. However, as scientific knowledge, government responses, and health policy related to COVID-19 shifted over time, the study team opted to reanalyze the data in light of these developments to decenter the immediate impacts of the pandemic and focus more broadly on how the pandemic intensified health inequities and shaped the provision of iOAT care. Further, while there were common questions and themes across all three interview waves, the pandemic-related content tended to shift in focus over time to the most recent or pressing COVID-19-related issues. For instance, interviews in April 2020 focused on immediate clinic changes in the first weeks of the pandemic, while interviews conducted in February 2021 explored participants’ future treatment plans as the COVID-19 vaccine was released. Thus, in this analysis, the study team decided to analyze themes emergent across the dataset, rather than focus on specific pandemic changes in each wave.

In the current analysis, data were analyzed using a constructivist and relativist lens, an orientation that assumes multiple and sometimes conflicting social realities. Through this approach, analysis underscores that knowledge is created in the interactions between interviewer and participants and are shaped by individuals’ backgrounds and experiences, and thus perspectives are value-mediated [34, 35]. In addition, the study team approached the qualitative analysis with an orientation to the social and structural features of clients’ lives and surrounding the iOAT clinic that informed their treatment experiences. In analyzing the interview transcripts, we used a multi-step flexible coding strategy [36]—a pragmatic, iterative approach to analysis that emphasizes abductive theory construction. As opposed to a purely inductive grounded theory method, this abductive analytical approach allows for the emergence of novel empirical findings through participant-generated avenues of inquiry, while incorporating existing theories and knowledge (e.g., pandemic impacts on substance use treatment disparities and overdose) [37]. First, using NVivo software, data were indexed into large “buckets” or overarching themes that mirrored the structure and sequence of the topic guide. Each set of indices was applied to transcripts from Waves A, B, and C, despite some shifts in COVID-19-related focus over time. The study team met to discuss this initial coded index and drew upon their collective expertise in iOAT research to prioritize relevant questions for focused coding and further inquiry. Next, relevant indices were parsed into more specific codes in targeted sections of the transcript, with a focus on how the pandemic impacted clients and their perceptions of iOAT treatment provision, as well as clinic challenges and opportunities that arose. Finally, NVivo tools (i.e., matrices) were used to validate findings and assess thematic saturation [36]. Following coding and team discussion, responses were synthesized within each major theme to generate representative findings in the results.

## Results

In total, we conducted 51 interviews with 18 iOAT clients, with ages that ranged from 17 to 71, with a median age of 53. There were eight men (44.4%) and 10 women (55.6%), with 11 (61.1%) participants identifying as White, five (27.8%) participants identifying as Indigenous, and two participants with unknown race/ethnicity. Demographics are listed in Table 1. In recounting their experiences, iOAT clients discussed not only the marked effects of the pandemic on the iOAT clinic and their clinic experiences, but also the secondary impacts of the pandemic on their lives and their communities. Clients described how the pandemic threatened their financial, physical, and mental wellbeing, which had indirect effects on their ability and willingness to access treatment. Like many health and social services, the pandemic constrained the iOAT clinic, but it also prompted novel approaches to treatment provision that ultimately reflected more flexible, patient-centered models of care.

### Pandemic impacts on the lives of iOAT clients

#### Economic (in)stability

Given the potential economic impacts of the pandemic, participants were asked whether their opportunities for income generation had changed and how these changes affected their financial stability. Most participants were receiving some form of government income assistance and noted that in response to the pandemic, the government had slightly increased this monthly rate. For some participants, this marginally higher income, when combined with reduced opportunity for spending due to pandemic restrictions, resulted in greater economic stability. For others, this additional increase in assistance only helped to offset the rising price of consumer goods amid pandemic supply chain interruptions. While few participants had full-time employment, some participants were engaged in informal or part-time work—opportunities that were generally reduced during the pandemic. As participant #17 remarked, “I’m having to stay home and not be on the street doing anything, I guess I am losing money.” For this participant and others, opportunities for odd jobs or street-based work (e.g., reselling goods; panhandling; informal recycling) were limited, but the informal nature of this work also precluded them from applying for pandemic relief benefits, such as the $2000 Canada Emergency Response Benefit (CERB). In several interviews, participants raised concerns about CERB recipients they perceived to be ineligible, and expressed fears about the consequences of an influx of money on their community (i.e., the “cheque effect”) [38, 39]. As participant #2 explained, “people are getting thousands and thousands of dollars get handed over to them through the government and people are just getting crazy.” For participant #7, who sold drugs to generate income, more disposable income in the community meant that he also received additional money: “I sell dope and I think it’s funny but I’ve seen more money now that I had before. Is it ‘cause everybody is getting more money? I don’t know, but I can’t complain personally.” Participant #9 felt this influx of money had shifted community dynamics and triggered negative emotions for him when traveling Downtown and to the clinic:

I don’t like to go down just for visits, because I do find that the temptation a bit unwieldy sometimes…People discovered this [Canadian Revenue Agency] benefit that everybody’s been getting…People have been living high on the hog Downtown in a way…It’s going to be really bad if people have to pay all that stuff back, right. There’s storm coming where that’s concerned. But anyway, no I don’t go Downtown—just to visit, only for when I need to.

As an iOAT client and employee, participant #18 explained how drastic fluctuations in income could impact clinic retention: “some people don’t come anymore. There’s a number of reasons for that and I couldn’t really nail them down, apart from the fact that there was money available for people and a lot of people applied for it and got it, so probably that would impact the numbers.” When asked if he was referencing the CERB, participant #18 replied, “That’s right, yeah, yeah. Lots of people have applied and got it. They’ll have to pay it back, I do believe, but nonetheless it’s there now for people and, you know, that’s a green light for a lot of folks.” In other words, participant #18 speculated that iOAT clients who received pandemic relief funds, a “green light,” may have spent their additional income on street-based drug supply, rather than visiting the clinic. While most participants did not explicitly point to a direct connection between their economic stability and retention in iOAT, the pandemic and associated government response had indirect impacts by generating economic uncertainty and by facilitating dramatic fluctuations in the local economy in which participants lived and worked.

#### Physical health

While the COVID-19 virus poses a health threat to everyone, those who are immunocompromised or have significant health issues face even greater health risks. IOAT clients emphasized that their underlying health problems, including HIV, Hepatitis C, cellulitis, cancer, and respiratory conditions, increased their risk of severe outcomes from COVID-19. Participant #12 expressed a common sentiment among participants: “I’m 66 years old, I have COPD and basically it’s end of story if I come in with [COVID-19]. I’m pretty much guaranteed to be a dead man.” For some participants, the increased risk of severe COVID-19 outcomes deterred them from seeking healthcare when needed. Participant #11 recounted her situation: “I thought I was having a heart attack ‘cause my arm went numb and I was getting chest pain, but I was so scared of going into emergency because that’s where COVID-19 [is].” Participant #14 described a similar circumstance: “I was terrified, I didn’t even want to come [to the hospital]. I almost made myself get sicker before not coming, because I was so terrified to come to the hospital because they were saying that’s how you catch COVID, in the areas like the hospital.” This reluctance to seek medical care was particularly salient at the start of the pandemic, amidst the highest levels of uncertainty and closures or restrictions on medical services, but anxieties around COVID-19 persisted throughout the duration of the study, suggesting enduring unmet treatment needs among clients.

These perceptions of heightened COVID-19 risk could also deter participants from accessing the iOAT clinic because of the perceived risk of exposure while in transit or within the clinic. Participant #15 remarked on her desire to switch treatment: “It’s way more stressful than before because I’ve got COPD, I have asthma so it’s—I honestly gotten terribly [scared] to leave the house…I’ve been having panic attacks. I'd like to actually switch to having my dose delivered from the clinic because I can’t handle the stress of coming up here.” For some participants, the risk of COVID-19 exposure was enough to prompt them to reduce the number of times they attended the clinic, in consultation with their doctor. Participant #4 explained why he reduced his iOAT visits to once per day, “Because of the pandemic. That as well as weather.” At the time of his last interview, he had not received a take-home dose and because of that, he “still wake[s] up sick most days,” and compensates with unregulated opioids. Some participants opted to change their treatment plan entirely due to the risk of exposure to COVID-19. As participant #10 explained, “I’m staying home as opposed to going to get my shot. I went on methadone until this is over. If I got sick, I don’t think that would be very good for me. I have COPD and other underlying problems, that wouldn’t be good if I got COVID.” When asked how the methadone treatment was going, participant #10 reiterated her desire to reduce her COVID-19 risk: “It keeps me at home so, you know? It was the only way that I could think of really, as opposed to going to the clinic. They don't practice safe anything around [here], in this [Downtown Eastside neighbourhood]. Not really.” As these perspectives highlight, many iOAT clients had to weigh the risk of exposure to COVID-19 against the risks of altering their treatment plan, which for some participants, like participant #4, meant unfulfilled treatment needs and subsequent exposure to unregulated opioids.

In addition to physical health risks, some iOAT clients experienced physical barriers to accessing services during the early pandemic response. Pandemic policies forced changes in occupancy for retail and health services, requiring people to wait in long lines outdoors. IOAT clients with physical disabilities remarked on how these changes impacted the services they were willing or able to access. Participant #13 described her situation: “I miss the food banks mostly. And in grocery stores you can’t even go in them really…Seems everywhere you go, you stand in line. …It’s difficult. I can’t do anything out here. I’m in a scooter too so that makes it even worse.” Policy changes around occupancy guidelines extended to the iOAT clinic setting, where clients had to wait outside before they could access their medication. For people with mobility issues, this waiting period could pose a challenge. As participant #2 explained: “Being a disabled person, I find it a little difficult sometimes to stand and the fact that [the clinic] is only allowed 8 clients in at any time, that’s added time to the wait.” Even when clients were able to stand in line outside of the clinic, some felt uneasy being in close proximity to others in a pandemic. Participant #3 explained the process of lining up to enter the clinic:

They all crowd around the door and want to be seen by the nurse, and [the nurse] writes down the names. So, I didn’t want to do that. I wanted to do the safe distance thing and so I keep away. And I wouldn’t get—like I missed two times. I’d have to phone them and tell them that I’m out here. I’m over about ten feet away from the crowd and then they would put me down [on the list].

Though iOAT clinic staff accommodated participant #3’s request for distance while waiting, pandemic conditions created physical challenges for iOAT clients and consequently, a physical barrier to treatment.

#### Mental health

In addition to anxiety about physical health risks, the pandemic negatively impacted iOAT clients’ mental health, largely by severing their social ties and reducing social support. In accordance with public health guidelines, many iOAT clients opted to reduce the number of social contacts or were forced to through visitor restrictions imposed by their social or supportive housing units. Without regular interaction from friends or family, participants described feeling bored or lonely, like participant #14, who expressed, “I don’t spend my time with a lot of people but since the COVID virus, I’ve been extremely lonely. It’s extremely hard to be home all alone all the time. You know, my doctor advised me to stay home as much as I could and to just stay away from everybody and just be home, that’s extremely hard.” For iOAT clients, feelings of loneliness and uncertainty around the pandemic were heightened by the concurrent overdose crisis facing their community. As participant #2 stated, “fentanyl is a pandemic and it's not being addressed quick enough.” Participant #8 described how these dual crises intensified her feelings of loneliness:

I used to have a lot of friends down here and now I don’t and I’m really lonely. I don’t really feel like befriending any more people down here because they’re just going to die or they’re going to do something to break my heart. I just don’t feel like making more friends. In that case, why be here? So I can sit here in my isolation, even without COVID? Like sleep and read my life away?

As participant #8 highlights, the isolating nature of the pandemic could amplify the mental health impacts of the overdose crisis, prompting more intense feelings of grief, loneliness, and worry. However, it is important to acknowledge the continuity of iOAT services during the pandemic and the clinic’s role in supporting clients’ health, as echoed by participant #7: “I say the clinic’s still open and that basically is my number one concern in my life. As long as that place stays open, I’ll stay alive, I know that.”

While participants were grateful for iOAT and the clinic, the mental health challenges experienced by iOAT clients during the pandemic could indirectly impact their treatment experiences. Participant #5 was accustomed to seeing her boyfriend regularly, who was also an iOAT client, and they would attend clinic together. However, when participant #5 couldn’t see her boyfriend due to pandemic restrictions in social housing, she felt “lonely and isolated and angry” and that “everything came to a halt when I couldn’t see him.” As a result, she stopped attending clinic altogether. With the attenuation of restrictions, participant #5 was able to see her boyfriend again: “he came back and got me [to go to clinic] and now we’re staying together again and things are better, but if it wasn’t for that, I wouldn’t have come back [to the clinic].” While participant #5 and other iOAT clients were eventually able to reconnect with local friends and family, a few participants remarked on how the pandemic prompted a desire to connect with family who lived further away, but that they felt unable to deviate from the clinic schedule. Participant #8 described her challenges around the routine:

I’ve been there for a long time and it’s just—I think I just need a break. I’ve gotten to a point where it’s too monotonous, too repetitive, too much of my time. I can’t do anything else. [There’s] no room to do anything else in the day, basically. I can’t go to [name of town] to go see my mom. By the time I get out there, I have to turn around and come back. I get to visit for an hour or two.

As a long-term client, participant #8 felt treatment was effective, but that the rigid schedule had a social cost, which was reinforced in the pandemic amidst the desire to reconnect with family. Taken together, interview data underscores that in addition to the general anxiety around the threat of COVID-19, iOAT clients were simultaneously facing rising rates of overdose in their community, disrupted social ties within their neighborhood, and the constraints of a strict treatment regimen—all of which had bearing upon their mental health.

### Pandemic impacts on iOAT treatment provision

As the iOAT clinic adapted to COVID-19 protocols, the most noteworthy changes for clients were the reduced clinic occupancy and wait times for entry. Some clients found it challenging to schedule their day around a clinic visit, arriving on time only to wait in line. Participant #1 recalls, “it’s a lot of chaos coming into the clinic every day because only certain amount of people [are allowed in] and sometimes I’d be dope sick and I just really didn’t wanna deal with people’s shit so I would just leave and then go buy dope and not go get my shot, which is not good at all.” For participants experiencing dope sickness or other obligations, these delays could be enough to deter participants from accessing treatment. Once inside the clinic, however, some participants were pleased with the new procedures, like when the post-medication assessment period was reduced from 15 to 5 min, to reduce risk of COVID-19 exposure. Participant #7 described this streamlined process: “I go in, sanitize my hands, they ask some questions, check-up, get meds, get my shot, wait for checkup and leave—it’s beautiful. I mean, 15 min, tops.” However, in trading the more relaxed clinic routines for efficiency, clients also observed that the general atmosphere had shifted at the clinic. Participant #17 spoke on the drawbacks of forgoing time in the post-assessment waiting room: “For some people, they don’t have a place to sit and relax and enjoy their meal or their coffee in the morning, or any time of the day. So, when they go in there, they can’t sit there after they get their shot and enjoy their meal, or their beverage.” Similarly, participant #8 described the clinic as “a lot more cold and a lot more—almost like a hospital ward.” Several clients also remarked on the closure of the clinic restroom, in part, to alleviate the burden of cleaning and monitoring by overextended staff. However, the loss of access to a clean restroom was noteworthy, especially for clients without housing. Participant #18 described this challenge for unhoused clients: “for people living outside, there are very few [restrooms] that are open. Most of them are packed, locked up, and not available to anybody and that’s not good for people.” While changes to clinic processes ensured that all clients could receive treatment amidst pandemic restrictions, the increased efficiency also meant the sacrifice of small comforts that drew clients to the clinic.

Changes in iOAT clinic processes also had consequences for the social dynamics within the clinic. Several participants described the clinic as a source of social engagement and community before the pandemic. Participant #14 expressed this sentiment: “I feel like I’m part of iOAT and I feel like they’re my family.” Yet, new pandemic protocols meant clients spent less time in clinic and more physically distanced, effectively reducing social engagement. Participant #8 described her experience with the changing social dynamic: “Everybody is distanced socially from people, [the clinic] doesn’t seem as close knit as it used to be. Pretty sad because it was pretty much the only place I feel like I could go to get any kind of experience with other people.” One clinic nurse (nurse #1) recalled similar feedback from a recent client survey regarding a new proposed clinic site:

When we did some client surveys about the new site, one of the big things people are asking for is a space for somebody could sit down for a few minutes or just hang out and review a magazine or whatever…And that’s something that I’ve heard as well, that we’re missing that kind of sense of community and I think it’s maybe not been said, but it is kind of tough cause all of our staff are wearing masks so we’re kind of losing that personal touch as well.

In addition to social engagements with friends and family outside the clinic, these perspectives highlight how the pandemic and related policies could shift dynamics within the clinic and compromise the long-standing social relationships among iOAT clients and staff.

### New opportunities in iOAT treatment provision

While the pandemic restricted clinic processes in some ways, it also accelerated the development of more flexible medication guidelines, in which clients had more autonomy around their health and medication regimen. To reduce iOAT clients’ health risks during the pandemic, providers worked with eligible clients to devise more flexible treatment plans, such as mixed treatment models, where clients could receive one injectable dose at the clinic and one take-home dose of oral medication (e.g., slow-release oral morphine; hydromorphone). Participant #11 recalled how she had been taking oral medications for over a year and when the pandemic began, staff reached out to her about transitioning to home delivery of medication (i.e., slow-release oral morphine). She had severe emphysema and was worried about going to the clinic but felt relieved when offered delivery: “The fact that I was given the opportunity to self-isolate and still be able to get what I need to keep me well is amazing. I really have to give the highest regards to the program…it’s life-changing.” Other clients were able to get longer prescriptions so they could reduce the frequency of clinic visits. Participant #9 was living in a different part of the city and felt that visiting Downtown so often was emotionally and physically difficult. However, during the pandemic, in response to his doctor increasing his prescription length, he remarked: “I was pretty happy about that…As much as I might miss the people [at clinic], I don’t like the idea of going down.” The opportunity for more flexible care prompted clients and staff to reimagine the possibilities for iOAT, with the hope that this flexibility would endure after the pandemic. When asked about desired changes for iOAT treatment, participant #2 responded, “It would be so much easier for me if I could get a morning carry shot, then I could go off to work. I mean, I’ve got to go to three places before I can even go to work. And by the end of the day, I've done that three times and it takes a big chunk of your day.” One clinic nurse (nurse #1) explained the novelty of some of these changes:

With iOAT being such a specialized service and kind of essential service for people, we’ve had to really modify all of our policies and procedures. Almost every aspect of the clinic has kind of adjusted... One of the new things that we’re doing is we’re actually outreaching DAM [diacetylmorphine] to clients who are isolating for COVID-19. So that’s a big, big, big change. We’ve never been able to take DAM off-site to somebody before.

As this iOAT nurse highlights, new procedures meant that clients with COVID-19 or isolating due to exposure were able to receive their medication safely outside of the clinic. While this nurse later acknowledged such personalized interventions may not be scalable, interview participants felt the flexibility to meet clients’ individual needs and have autonomy over their medication regimen was a critical characteristic of the program and hoped these changes would endure.

In addition to facilitating more patient-centered care, the pandemic highlighted important moments of connection and communication of health information between iOAT clients and staff. Participants felt connecting with staff at the clinic was a beneficial part of their treatment routine and this interaction seemed to provide a sense of community and stability amidst pandemic uncertainty. Participant #1 described her experience with staff: “when I come to the clinic, they say hi to me, ‘how are you?’ If they notice that I’m acting strange or not in the best mood, they come and ask me how I am and I can tell they actually care. And then when…shitty stuff goes on for me, sometimes they go out of their way and help me out.” In addition to emotional support, participants highlighted the role of iOAT staff in providing relevant information related to COVID-19 as well as in helping clients reduce their health risks. Participant #15 observed, “the place is being kept spotless and everybody—they're given the hand sanitizer at the door and making people wear masks and what have you. I think they’re doing everything they can do.” Clinic staff were even able to facilitate vaccination for clients who were medically vulnerable and thus prioritized in the province’s vaccine rollout. Participant #16 recalled the phone call that he and his partner received from clinic staff:

They said basically like ‘we’ve got [the vaccine], you guys should get up here.’ [My partner] and I looked at each other and said, ‘let’s get our ass up there.’ We got ready in about 15 minutes. Got on the bus got up there about like 25 minutes later. That was just good luck. It was one of the nurses in one of the other programs, and they recognized [my partner’s] name because she had COPD.

Though not all clients had received a vaccine at the time of interview, iOAT staff may have encouraged vaccine uptake, by directing clients to vaccination sites or by sharing their own vaccine experiences. In sharing his vaccine perspective, participant #9 remarked, “I’d be willing to use a vaccine if it meant giving me more freedom to move about in the world. A lot of doctors said it was safe, I trust them.” Like participant #9, many of the clients trusted the medical opinions of clinic staff, highlighting how iOAT treatment engagement could be an effective pathway for communication about the COVID-19 pandemic.

## Discussion

The purpose of this qualitative study was to explore how the COVID-19 pandemic impacted the lives of iOAT clients and their perceptions of and experiences with iOAT treatment during dual public health crises. In describing the impacts of the pandemic, participants remarked on their financial instability, as their opportunities for low-threshold, income-generating activities dwindled. While government income assistance rates slightly increased, most participants were not eligible to receive the more substantial Canada Emergency Response Benefit, and worried about an influx of pandemic financial relief on the community and the consequences for the increasingly unstable, local drug market. Participants were also concerned about their COVID-19 health risks, as many were immunocompromised or faced respiratory issues, and had to weigh the benefits of receiving treatment or accessing other services with the inherent risks of exposure. When asked about their mental health, participants described feelings of loneliness and isolation, as they were physically distanced from family and friends, and as supportive housing restrictions limited their social interactions. These mental health impacts were amplified by the ongoing drug poisoning crisis, and participants drew attention to the continual rise of overdose deaths in their community.

Our findings also revealed how the pandemic directly impacted the iOAT clinic and treatment provision. While the iOAT clinic remained open throughout the pandemic, clinic staff had to navigate their own uncertainty and fear, while accommodating provincial safety guidelines and developing new procedures to reduce exposure, such as restricting clinic occupancy, moving the clinic queue outdoors, closing the restroom, and limiting clients’ time in the clinic. For some clients, this increased efficiency meant less time interacting with clinic staff and other clients—interactions that were a valued component of clinic visits. Still, the clinic remained an important touchpoint for clients, who received both social connection and critical information about COVID-19 risk mitigation from staff. Some iOAT clients appreciated the streamlined clinic processes which, along with greater flexibility in dispensation of take-home oral doses, could be liberating for clients. IOAT client narratives reflected an increased sense of trust and autonomy, in which the clinic staff and clients worked together to identify and plan for their individual treatment needs. When combined with sustained and meaningful staff-client interaction, the continuation of this patient-centered model of care could have benefits for clients’ physical and emotional wellbeing, as well as retention in care.

In communities around the world, the COVID-19 pandemic laid bare underlying social inequities [40], and as reiterated by our findings, these inequities had notable consequences for the wellbeing of PWUD and their treatment experiences. First, for PWUD experiencing socioeconomic marginalization or unemployment, financial instability can not only jeopardize their material security, but also impact substance use patterns, drug scene involvement, and exposure to overdose risks, criminalization, or violence [41–43]. Second, COVID-19 threatened the physical health of iOAT clients, and ongoing research has demonstrated how substance use and comorbidities position PWUD for greater risk of severe COVID-19 outcomes [44, 45]. As a result, some clients had to weigh the risk of COVID-19 exposure, sometimes multiple times per day, against the risk of sourcing unregulated opioids and potential overdose. Third, while the pandemic had significant consequences for mental health globally, PWUD may have fewer social supports to draw upon, due to drug-related stigma and discrimination from friends or family [46–48]. For iOAT clients with friendships at the clinic or through supportive housing, these social connections were severed by institutional policies that prioritized physical distancing. While such pandemic policy decisions are complex, our data underscores how these restrictions on social connection and to personal autonomy can have substantial mental health impacts that should be seriously considered. Finally, clients’ perspectives on the challenges associated with daily iOAT clinic visits are consistent with previous research [18], but as the pandemic progressed, the clinic sought to reduce these barriers to care and mitigate both overdose and COVID-19 risk. Eligible iOAT clients were offered individualized and flexible medication dispensation that eased their treatment burden and barriers to adherence, and fostered an environment of respect for patient autonomy and shared decision-making [29].

### Implications for policy and practice

These findings offer key insights for providers and policymakers in supporting PWUD and their substance use and treatment goals. Given the impacts of financial stability on treatment experiences, policymakers should strive to create low-threshold, income-generating activities for PWUD. Meeting clients’ accessibility needs (e.g., accommodate mental and physical health needs; time flexibility for health/treatment visits) is imperative for these opportunities to support their treatment goals and engagement. Such opportunities may provide additional income as well as foster social connections and intrinsic meaning. In addition to work-related social connections, PWUD living alone or with little social support may derive benefit from social interactions within the substance use treatment setting, as highlighted by iOAT participants. In recognizing the health benefits of social connection, local governments could also provide opportunities for interaction within neighborhoods (e.g., community events, accessible public spaces), beyond those mediated by institutions. Finally, substance use treatment providers could seek to increase flexibility and client autonomy where possible in working with clients’ self-identified goals and devising substance use treatment plans. For some clinics, this could mean offering extended hours to accommodate work and caregiving schedules. In other clinics, more flexible medication dosing regulations would allow physicians to respond to client needs more effectively. Depending on local treatment policies and community accessibility needs that account for cultural and geographical differences, it may be possible to offer telehealth visits or home delivery of medications to those with transportation barriers or mobility challenges.

### Limitations

This analysis has some limitations. First, there may be potential for response bias, including courtesy bias in reporting negative perceptions of the iOAT clinic, or social desirability bias in underreporting substance use patterns or criminalized activity. However, interviewers established relationships with clients, and sought to distinguish themselves as separate from clinic staff in an effort for reassure participants that their individual responses would not be shared with the clinic. Second, due to social distancing protocols, interviews were conducted over the phone instead of in person. However, phone interviews may have allowed for greater flexibility in timing for client interviews and certainly reduced risks of COVID-19 exposure. Finally, there were some participants who were lost to follow-up and unable to be interviewed at all three timepoints.

While iOAT is not available in all jurisdictions, these findings have broader relevance for addressing the unmet economic, social, health, and substance use treatment needs of PWUD and the promotion of health equity through dual public health crises. In this study, the iOAT clinic was able to maintain patient-centered care and pursue more flexible, individual approaches to substance use treatment, even amid uncertainty and change. Regardless of treatment modality, pandemic-era changes that reflect progress toward patient-centered models of care for substance use disorders should be made permanent [49], and reflect recommendations for equitable pandemic recovery [50].

## Data Availability

The datasets generated and/or analyzed during the current study are not publicly available due to the qualitative design that risks potential reidentification as well as to protect the privacy of clinical patient data, but a modified version of the data may be available from the corresponding author on reasonable request.

## Abbreviations

CERB: Canada Emergency Response Benefit
iOAT: Injectable opioid agonist treatment
OAT: Opioid agonist treatment
PWUD: People who use drugs
